# Cardiovascular Disease and Hemodialysis

**DOI:** 10.3390/medicina61101796

**Published:** 2025-10-05

**Authors:** Keren Cohen-Hagai

**Affiliations:** 1 Department of Nephrology and Hypertension, Meir Medical Center, Kfar Saba 4428164, Israel; keren.cohen@clalit.org.il; 2 Gray Faculty of Medical and Health Sciences, Tel Aviv University, Tel Aviv 69978, Israel

Cardiovascular disease (CVD) remains the leading cause of morbidity and mortality in patients undergoing maintenance hemodialysis (HD). The interplay between uremic toxins, vascular calcification, chronic inflammation, malnutrition, and traditional cardiovascular risk factors contributes to a disproportionately high burden of cardiovascular morbidity and mortality in HD population [1,2,3,4,5]. Beyond the clinical implications, this burden carries significant economic consequences for healthcare systems [6]. The persistently elevated and in many settings unacceptable mortality rates in this population reflect not only the complexity of cardiovascular disease pathogenesis in uremia, but also the inadequacy of current preventive and therapeutic strategies [1,2,3,4,5]. These unique pathogenic mechanisms demand focused research efforts and tailored interventions to improve outcomes in this vulnerable group.

This Special Issue of *Medicina*, “Cardiovascular Disease and Hemodialysis”, brings together original clinical research that deepens our understanding of the multifaceted cardiovascular burden in this population. The selected studies provide important insights into risk stratification, predictive biomarkers, treatment outcomes, and the pathophysiology of CVD in HD patients.

Nakić et al. evaluated the role of conventional cardiovascular risk factors in dialysis patients with coronary artery disease. Despite the altered metabolic milieu in end-stage kidney disease (ESKD), traditional risk factors such as hypertension, diabetes, and dyslipidemia remained significant contributors to coronary artery disease. This reinforces the relevance of guideline-directed cardiovascular risk factor management in HD patients [7].

LDL cholesterol represents a conventional cardiovascular risk factor that is also addressed in this issue. A retrospective cohort study evaluated the prognostic significance of LDL-C in HD patients hospitalized with acute coronary syndrome (ACS) [8]. Despite similar LDL-C levels and statin use across groups, 30-day and 1-year mortality were significantly higher in HD patients compared to controls. Survival correlated not with LDL-C levels, but with markers of nutrition and inflammation. These findings suggest that in HD patients, inflammation and malnutrition outweigh LDL-C as determinants of post-ACS prognosis [8].

In line with evidence that malnutrition and inflammation outweigh traditional risk factors, Dragoș et al. demonstrated that inflammatory pathways—not solely passive mineral deposition—drive vascular calcification in uremia. This underscores the importance of anti-inflammatory strategies as potential targets in cardiovascular risk mitigation [9]. In ESKD patients, intravascular calcification is both a contributor to and a consequence of CVD. A retrospective study in HD patients found that over 90% had intracranial arterial calcifications (ICACs), markedly higher than among controls [10]. ICAC severity correlated with hypoalbuminemia, hyperphosphatemia, and elevated CRP. In addition, higher ICAC scores independently predicted 1-year mortality, highlighting the potential of this measure as a prognostic imaging biomarker in this population.

Wu et al. demonstrated that serum endocan levels, a marker of endothelial activation and inflammation, were significantly associated with increased aortic stiffness in HD patients. The findings provide evidence linking endocan to vascular remodeling and support its utility as a surrogate marker of cardiovascular dysfunction in this group [11]. Šafer et al. explored the prognostic role of endocan in non-diabetic HD patients. Elevated serum endocan predicted adverse cardiovascular events and mortality, further supporting its emerging role as a biomarker of endothelial dysfunction and systemic risk in HD populations [12].

Endothelial dysfunction drives both cardiovascular disease and vascular access complications, both of which significantly affect the prognosis of HD patients. Venegas-Ramírez et al. analyzed long-term survival outcomes in relation to vascular access type. As expected, arteriovenous fistulas (AVFs) were associated with better outcomes compared to catheters. Despite this, AVF utilization remains suboptimal, indicating the need for early referral, vascular mapping, and multidisciplinary planning [13].

The prospective, observational study by Domjanović Matetić et al. examined echocardiographic parameters in ESKD patients undergoing transition from HD to hemodiafiltration (HDF). They found that echocardiographic indices could serve as early markers of cardiovascular adaptation and might inform the timing and benefit of switching to HDF [14].

Dimitrijevic et al. conducted a 24-month cohort study examining bleeding and thrombotic complications in HD patients with atrial fibrillation. Results revealed high rates of both hemorrhagic and thromboembolic events, reflecting the complex and competing risks in this population. These findings call for individualized, risk-adapted antithrombotic strategies for dialysis patients with atrial fibrillation [15].

The studies featured in this Special Issue focusing on cardiovascular disease and hemodialysis collectively emphasize that cardiovascular risk in HD patients is multifactorial, involving not only traditional risk factors but also inflammation, malnutrition, vascular access characteristics, and endothelial dysfunction. Novel biomarkers such as endocan, along with imaging features like ICAC, may aid in refining cardiovascular risk assessment and support evaluating the role of advanced technological options in HD, such as HDF, in controlling and mitigating these processes. Ongoing research is essential to better tailor prevention and treatment strategies to improve survival and quality of life in this vulnerable population.
